# Extremely high infiltration of CD8^+^PD-L1^+^ cells detected in a stage III non-small cell lung cancer patient exhibiting hyperprogression during anti-PD-L1 immunotherapy after chemoradiation: A case report

**DOI:** 10.3389/fonc.2022.969493

**Published:** 2022-12-02

**Authors:** Changshun Wu, Kai Zhou, Yan Zheng, Dongxiao Lv, Miaoqing Zhao, Yue Hu, Fei Qi, Xin Wang, Hong Feng

**Affiliations:** ^1^ Department of Surgery, Shandong Provincial Hospital Affiliated to Shandong First Medical University, Jinan, Shandong, China; ^2^ Department of Surgery, Shandong Provincial Hospital, Cheeloo College of Medicine, Shandong University, Jinan, Shandong, China; ^3^ Cancer Center, Shandong Provincial Hospital Affiliated to Shandong First Medical University, Jinan, Shandong, China; ^4^ Cancer Center, Shandong Provincial Hospital, Cheeloo College of Medicine, Shandong University, Jinan, Shandong, China; ^5^ Department of Outpatient, Shandong Provincial Hospital Affiliated to Shandong First Medical University, Jinan, Shandong, China; ^6^ Department of Pathology, Shandong Provincial Hospital Affiliated to Shandong First Medical University, Jinan, Shandong, China; ^7^ Department of Oncology, The Central Hospital of LaiWu, Jinan, Shandong, China

**Keywords:** non-small cell lung cancer, CD8^+^PD-L1^+^, hyperprogressive disease, PD-L1, durvalumab

## Abstract

In recent years, immune checkpoint inhibitors (ICIs), represented by PD-1/PD-L1 monoclonal antibodies, have become a research hotspot in the field of oncology treatment. Immunotherapy has shown significant survival advantages in a variety of solid tumors. However, the phenomenon of hyperprogressive disease (HPD) in some patients treated with immunotherapy is gradually getting more attention and focus. An early understanding of the characteristics of HPD is crucial to optimize the treatment strategy. We report a patient with unresectable stage III lung adenocarcinoma who developed HPD with metastasis during consolidation therapy with durvalumab after chemoradiation. To further investigate the potential mechanism of HPD after anti-PD-L1 treatment, primary lung baseline tissue, baseline plasma, post-immunotherapy plasma, and liver metastasis samples of the patient were detected *via* next-generation sequencing (NGS). Then, multiplex immunohistochemistry (mIHC) was performed on primary lung baseline tissue and liver metastasis samples. *KRAS* and *p.G12C* were identified as the major driver mutation genes. With a low tumor mutation burden (TMB) value, the patient presented a very high percentage of CD8^+^PD-L1^+^ T cells that infiltrated in the baseline tissue, with 95.5% of all CD8+ cells expressing PD-L1 and a low percentage of CD8+ T cells expressing PD-1. After the emergence of HPD from immunotherapy, liver metastases were similarly infiltrated with an extremely high proportion of CD8^+^PD-L1^+^ T cells, with 85.6% of all CD8+ cells expressing PD-L1 and almost no CD8+ T cells expressing PD-1. The extreme infiltration of PD-L1^+^CD8^+^ T cells in the tumor microenvironment of baseline tissue might be associated with the aggressive tumor growth observed in anti-PD-L1 treatment for related HPD and could be a potential biomarker for HPD development.

## Introduction

In the era of immuno-oncology, the use of inhibitors such as programmed cell death 1 (PD-1) and programmed cell death ligand 1 (PD-L1) significantly improved the survival in patients with a variety of solid tumors (1–3), but it was associated with a unique series of immunotherapy-related adverse events, including hyperprogressive disease (HPD). Studies showed that PD-1/PD-L1 blockade can lead to HPD (4). HPD is mainly characterized by increased lesion size and more rapid progression of the lesion after immunotherapy (5). The incidence of HPD varies among different solid tumors (6, 7), with about 14% in non-small cell lung cancer patients. Immunotherapy-associated HPD usually indicates a poor clinical prognosis, which has drawn great attention from clinicians. It has become an urgent issue on how to reduce the damage caused by HPD to patients. The predictors of HPD are in the preliminary stage of study. Research showed that MDM2/4 amplification (8), EGFR mutations (9–11), and advanced age (12) may be the risk factors for HPD. However, the underlying mechanisms and biomarkers of HPD remain unclear. In this case, we report a stage III lung cancer patient who developed HPD during consolidation therapy with durvalumab after chemoradiation. To further discuss the HPD phenomenon associated with anti-PD-L1 therapy, we examined the genomic and immune microenvironmental features to explore the causes of HPD development in the patient and the potential biomarkers that may predict the development of HPD.

## Case description

**Figure 1:**
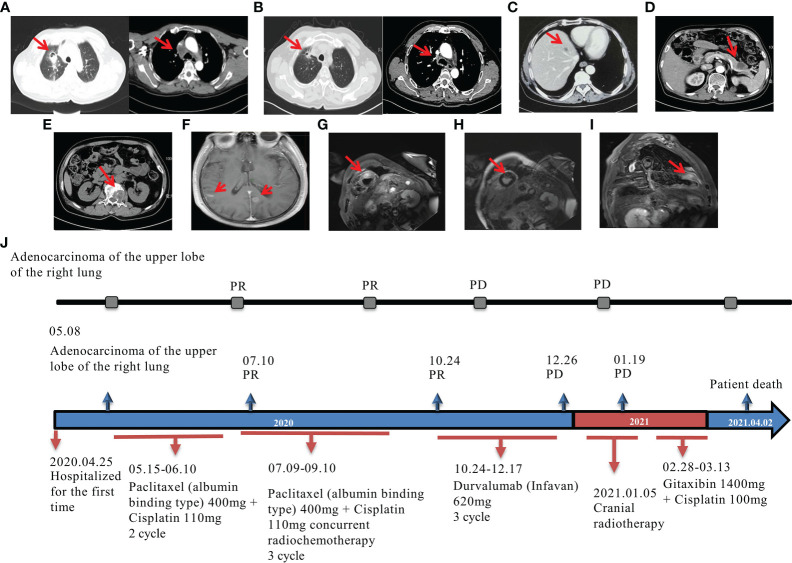
Imaging data of the patient during treatment. CT examination of the chest showed partial response (PR) after chemoradiotherapy. **(A)** The initial CT scan of the patient demonstrated a lesion in the right upper lobe of the lung with enlarged right mediastinal lymph nodes and extracapsular invasion; **(B)** CT scan showed PR of the lesions 1 month after chemoradiation; multiple metastases arising after the use of durvalumab demonstrating a hyperprogressive disease. CT images show **(C)** liver metastasis, **(D)** pancreatic metastasis, and **(E)** lumbar metastasis. MR images show **(F)** brain and meningeal metastases and **(G–I)** myocardial metastases. **(J)** Patient treatment chart.

A 62-year-old Chinese male patient visited the Central Hospital of LaiWu on 25 April 2020, who had cough for a month and blood in the sputum without an apparent trigger and had no improvement with cough suppressants. The patient had a history of smoking but had quit for 23 years. There was no family history of malignant tumors. Physical examination revealed no obvious abnormalities. Chest CT showed a lesion in the right upper lobe of the lung with enlarged right mediastinal lymph nodes and extracapsular invasion. Lung puncture biopsy revealed a poorly differentiated lung adenocarcinoma, which was T2aN2M0 (stage IIIA) according to the 2017 edition of the AJCC staging (**Figure 1A**). After being given tranexamic acid and other drugs to stop the bleeding, the patient’s condition improved. For further treatment, the patient was referred to Shandong Provincial Hospital. After a multidisciplinary consultation, the patient was evaluated as unresectable stage III NSCLC. Concurrent chemoradiotherapy and consolidation with durvalumab was recommended. From 15 May 2020 to 10 September 2020, the patient was given albumin-bound paclitaxel (400 mg) combined with cisplatin (110 mg) every 3 weeks and two cycles of inductive chemotherapy and three cycles of concurrent chemoradiotherapy with 60 Gy/30f to the primary tumor and metastatic lymph nodes. CT re-examination showed good changes in the right lung cancer and mediastinal lymph nodes after treatment, and the patient had a partial response (PR) according to RECIST criteria 1.1 (**Figure 1B**).

On 24 October 2020, 1 month after radiotherapy, the patient started to use durvalumab (620 mg) every 2 weeks. After three cycles of immunotherapy, the patient presented with chest tightness and breathlessness and low back pain. Systemic re-examinations showed liver metastases, pancreatic metastases, and multiple intra-abdominal metastases (**Figures 1C, D**). Magnetic resonance imaging (MRI) results of the head and spine showed multiple spinal metastases in the bone (**Figure 1E**) and brain and meningeal metastases in the central nervous system (**Figure 1F**). Cardiac MRI and ultrasound findings showed very rare myocardial metastases (**Figures 1G–I**). The overall treatment process diagram is shown in **Figure 1J**. In addition, chest CT showed increased inflammation in the right lung, with possible radiation pneumonia. To distinguish between pseudoprogression and HPD, a biopsy of the liver lesion was made and metastatic cancer was detected. Due to the advanced progression of the disease, durvalumab was withheld, and palliative irradiation to the brain and bone metastases was performed. The adherence of this patient was good during treatment. Unfortunately, the patient died of tumor progression after approximately 1 month of continued chemotherapy with gemcitabine (1,400 mg) and cisplatin (100 mg) every 3 weeks.

### Patient perspective

“Throughout my clinical care, I was informed about the diagnosis of my disease, the risks and benefits associated with treatment. After chemoradiotherapy, I felt relief from my cough and my strength returned. However, after 3 cycles of durvalumab immunotherapy, my cough got worse and I lost weight. Although the doctor later imposed local radiotherapy on me, by this time my strength was not what it used to be and I was confined to bed.”

### Profiling of genomic and local immune characteristics

**Figure 2:**
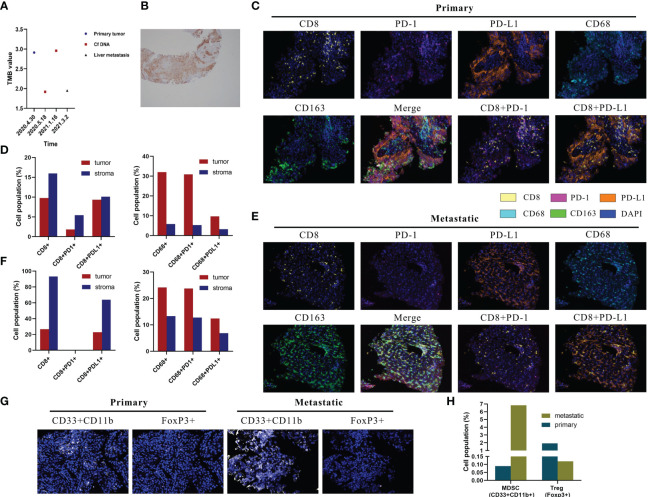
Multiplex immunohistochemistry (mIHC) results of lesion specimens and tumor mutation burden (TMB) and PD-L1 detection. **(A)** Dynamic changes in tumor mutation burden of the lung, blood, and liver. Definition of tissue tumor mutation burden (tTMB) and blood tumor mutation burden (bTMB): the TMB was defined as the number of somatic, coding, base substitutions, and indel mutations per megabases of the genome examined; bTMB and tTMB were calculated based on the candidate mutations according to the following formula: Absolute mutation count × 1,000,000/Panel exonic base num. **(B)** PD-L1 protein expression in paraffin sections of the primary lesion. **(C)** Representative images of CD8, CD68, PD-1, PD-L1, and CD163 shown by mIHC in the primary tumor. Nuclei (blue) were counterstained by DAPI. The original magnification was ×200. **(D)** Quantification results of mIHC of the primary lesion. **(E)** Representative images of CD8, CD68, PD-1, PD-L1, and CD163 shown by mIHC in liver metastatic tumors. Nuclei (blue) were counterstained by DAPI. The original magnification was ×200. **(F)** Quantification results of mIHC of liver metastases. **(G)** Representative images of CD33^+^CD11b^+^ and FoxP3^+^ in primary and liver metastatic tumors. Nuclei (blue) were counterstained by DAPI. The original magnification was ×200. **(H)** Quantification results of myeloid-derived suppressor cells and regulatory T cells.

Driver mutations associated with tumorigenesis were examined by next-generation sequencing (NGS). As shown in **Table 1**, *KRAS* and *G12C* were identified, with mutant allele frequency (MAF) of 53.93%, 0.53%, 7.41%, and 12.86% in the primary lung baseline tissue, baseline plasma, post-immunotherapy plasma, and liver metastasis samples. We observed that the *KRAS* and *p.G12C* mutation frequency in the plasma had significantly increased, 0.53% to 7.41%, after HPD. In addition, genetic testing showed that the patient’s plasma and tissue tumor mutational load (TMB) values have been very low (**Figure 2A**). In the primary lung baseline tissue, the expression level of PD-L1-positive cells accounted for 80% of the tumor cells (**Figure 2B**).

**Table 1:** Genomic alterations in specimens collected at different time points.

Timelines	Primary lung baseline tissue	Baseline plasma	Post-immunotherapy plasma	Liver metastasis tissue
Genomic alterations	FANCD2. c.1278+3_1278+6delAAGT ATM. c.618T>G TNFAIP3. c.1273C>G KRAS. c.34G>T MAP3K1. c.1485G>T MAX. c.31_32dupAG EPHA5. c.1964_1965delinsGG TP53. c.239dupC	IRS2. c.1082T>A KRAS. c.34G>T EPHA3. c.2764G>C	SUFU. c.670C>G TNFAIP3. c.1273C>G KRAS. c.34G>T MAX. c.31_32dupAG TP53. c.239dupC	FANCD2. c.1278+3_1278+6delAAGT TET2. c.2740C>G PIK3CG. c.2174G>C APC. c.4125_4127delinsTAG MET. c.1990C>G RAD21. c.59C>A RAD21. c.56T>C TNFAIP3. c.1273C>G HMCN1. c.15344A>T SF3B1. c.1896T>A SF3B1. c.1894T>C H3F3A. c.173C>T PALB2. c.398G>C KRAS. c.34G>T CDK8. c.938A>T KIT. c.2421_2422delinsTC MAX. c.31_32dupAG TP53. c.239dupC PTPRD. c.3506T>A EPHA7. c.475G>C DICER1. c.5532G>C

The local immune microenvironment was explored by multiplex immunohistochemistry (mIHC) assay using the Opal five-color IHC Kit (PerkinElmer, USA). We analyzed the multiple immune components in the primary lung baseline tissue and liver metastasis tissue samples, with the panels including CD8, PD-1, PD-L1, CD68, and CD163. As shown in **Figure 2C**, CD8^+^ lymphocytes and CD68^+^ macrophages were observed with high infiltration in the baseline tissue microenvironment. Quantitative analysis showed that CD8^+^ T cells, CD8^+^PD-L1^+^ T cells, and CD8^+^PD-1^+^ T cells accounted for 9.75%, 9.32%, and 1.81% and 15.95%, 10.11%, and 5.42% in the tumor and stroma regions, respectively (**Figure 2D**). This showed the high percentage of CD8^+^PD-L1^+^ T cells in the tumor region as 95.5% (9.32%/9.75%) of CD8^+^ cells expressed PD-L1. It is rare to see the extremely low proportion of CD8^+^PD-1^+^ T cells and the extreme infiltration of CD8^+^PD-L1^+^ T cells in the tumor microenvironment. Moreover, CD68^+^CD163^+^ accounted for 9.69% and 3.21% in the tumor and stroma regions (**Figure 2D**). Examination of the immune microenvironment in the liver metastasis revealed a high degree of CD8^+^ and CD68^+^ cell infiltration (**Figure 2E**). Quantitative analysis revealed that CD8^+^ T cells, CD8^+^PD-L1^+^ T cells, and CD8^+^PD-1^+^ T cells accounted for 26.74%, 22.89%, and 0% and 93.24%, 63.89%, and 0% in the tumor and stroma regions, respectively (**Figure 2F**). It could also be seen in **Figure 2F** that a high percentage of CD8^+^PD-L1^+^ T cells was infiltrated in the tumor region as 85.6% (22.89%/26.74%) of CD8^+^ cells expressed PD-L1 with nearly no CD8^+^PD-1^+^ T cells. Moreover, CD68^+^CD163^+^ accounted for 12.40% and 6.87% in the tumor and stroma regions (**Figure 2F**).

In addition, we explored the relevance of high PD-L1^+^CD8^+^ cell frequencies to immunosuppressive cells. Myeloid-derived suppressor cells (MDSCs) were defined as CD33^+^CD11b^+^, and regulatory T cells (Tregs) were defined as FoxP3^+^ cells. Notably, MDSC levels were significantly increased and Treg levels were markedly decreased in liver metastases than in the primary tumor (**Figures 2G, H**).

## Discussion

The PACIFIC trial shows that, compared with placebo, durvalumab consolidation therapy can improve the OS of patients with unresectable stage III NSCLC after concurrent chemoradiotherapy (13, 14). Concurrent chemoradiotherapy followed by further consolidation therapy with durvalumab is already the standard treatment modality for patients with unresectable stage III lung cancer (15, 16). In this patient’s primary lung tissue, immunohistochemical results showed a high expression of PD-L1. The NGS results identified *KRAS* and *G12C* mutations. These results suggested that the patient benefited from the treatment (17–19). Unfortunately, this case not only failed to obtain benefits but also had a malignant event (HPD). For this accident, we first reviewed the PACIFIC study and searched the literature directly related to therapy and found that there are few effective therapeutic markers for durvalumab consolidation therapy. In a case of stage IIIA NSCLC reported by Khreis et al., the patient also developed brain metastases after treatment with durvalumab and was diagnosed with HPD, while research on the mechanism has not been performed (20). Therefore, for the recommended consolidation immunotherapy after chemoradiotherapy, it is very necessary to study its therapeutic markers. Furthermore, it makes us think that when a sequential design-based therapy was implemented in clinical work and significant benefits occurred in the patient at a certain point during the sequential process, the doctor may seize the opportunity to cut into the next intervention plan, such as surgery, ablation, etc., or continue to perform sequential therapy. Based on the hint the case gave us, the contingencies in sequential therapy are worth pondering and studying. For the malignant event (HPD) here, we consider this to be an important and timely lesson learned.

We found that in the lung primary tumor baseline tissue of this patient, neither the infiltration level of CD8^+^PD-1^+^ cells nor that of CD8^+^PD-L1^+^ cells was on the cutoff side of the reported immune benefit (21–23). Simultaneously, we observed that the baseline tumor microenvironment of this case had a very high proportion of CD8^+^PD-L1^+^ T cells. An abnormal tumor microenvironment may be closely associated with HPD induced by immune checkpoint inhibitors (24). Is the extreme infiltration of PD-L1^+^CD8^+^ T cells in the tumor microenvironment a potential factor for HPD? Currently, although there is no direct clinical-level evidence about which CD8^+^PD-L1^+^ cells were involved in the process of anti-PD-L1 therapy for HPD, a study from George Miller’s group has some hints for us (25). *In-vitro* experiments showed that after treating mouse spleen-derived PD-L1^+^CD8^+^ T cells with αCD3/28 + IgG Fc and αCD3/28 + PD-L1 Fc, respectively, in the αCD3/28 + PD-L1 Fc-treated group, the expressions of Tbet, TNF-α, and LFA-1 were downregulated compared with those in the αCD3/28 + IgG Fc control group, which means that after the addition of anti-PD-L1 antibody *in vitro*, the transcriptional activators, effector cytokines, and activated surface markers of PD-L1^+^CD8^+^ T cells were significantly decreased, and the activation of CD8^+^ T cells was inhibited. Based on the results of this *in-vitro* cell experiment, we boldly speculate that the same phenomenon occurs in the human body. This case also supports our idea. After using durvalumab (anti-PD-L1 antibody), the patient not only had no tumor response but also developed HPD. In the baseline tumor microenvironment of this case, 95.5% of CD8^+^ cells express PD-L1, which is extremely rare. Combined with the previous *in-vitro* experimental results, this phenomenon is very important and should be the most likely cause of HPD. In this case, durvalumab may inhibit the activation of almost all CD8^+^ T cells in the tumor microenvironment, resulting in the decrease or incapacitation of the antitumor ability of CD8^+^ T cells, which further leads to rapid tumor progression and HPD. Further growth advantage of subpopulations of cells with major driver mutations, such as *KRAS* G12C, might be another reason for HPD (26). In addition, there were also some CD68^+^CD163^+^ suppressive macrophages in the tumor microenvironment of the baseline tissue. The study of Kim and colleagues showed that M2 macrophages (CD68^+^CD163^+^) are widely distributed in the stromal region of HPD patients and influenced by the tumor immune microenvironment (27). A recent study has shown that macrophages with M2 polarized can upregulate PD-L1 expression, subsequently leading to the decrease in CD8^+^ T-cell activity enabling immune escape and tumor growth (28). Whether CD68^+^CD163^+^ macrophages promote the occurrence of HPD needs an in-depth discussion. Moreover, in melanoma patients, it has been shown that a high frequency of PD-L1^+^CD8^+^ cells is associated with increased MDSCs and that there is a strong correlation with Tregs (29). We also examined the MDSC and Treg expressions in the primary and metastatic tumors of this non-small cell lung cancer patient, consistent with the trend presented in the melanoma patient.

However, our research has some limitations. First of all, we only used mIHC to identify cell subsets and their frequencies due to the lack of samples. The specific biological functions of CD8^+^PD-L1^+^ cells and PD-L1 signaling on immune cells require further studies. Secondly, we only observed one patient who developed HPD after using anti-PD-L1 with extreme infiltration of CD8^+^PD-L1^+^ T cells. So, the correlation between high infiltration of PD-L1^+^CD8^+^ T cells and clinical outcome remains to be explored in a larger cohort. In addition, since the patient did not receive a biopsy before durvalumab treatment, implying more treatments than just immunotherapy between the two available biopsies, we cannot exclude that some changes in the microenvironment may have occurred during these treatments: CD8^+^PD-L1^+^ cells were present in high levels already before the treatment with durvalumab or it is possible that they started to increase gradually during the treatment with paclitaxel + cisplatin and reach the peak just before durvalumab treatment or that there is the possibility that the increase of CD8^+^PD-L1^+^ cells started after durvalumab treatment. Further studies need to be performed to determine when CD8^+^PD-L1^+^ cells start to increase.

This report presents that the extremely high infiltration of CD8^+^PD-L1^+^ T cells may be a biomarker of HPD, which may provide a new perspective to explore the pathogenesis of immunotherapy related to HPD and to look for potential biomarkers.

## Data availability statement

The original contributions presented in the study are included in the article/supplementary material. Further inquiries can be directed to the corresponding author.

## Ethics statement

Written informed consent was obtained from the individual(s) for the publication of any potentially identifiable images or data included in this article.

## Author contributions

CW, KZ, and HF designed the study. Acquisition of data was done by YZ, DL, MZ, YH, FQ, and XW. Analysis and interpretation of data were done by CW, KZ, YZ, DL, YH, FQ, XW, and HF. Writing and revision of the manuscript were done by CW, KZ, and HF. Review was done by all authors. All authors contributed to the article and approved the submitted version.

## Conflict of interest

The authors declare that the research was conducted in the absence of any commercial or financial relationships that could be construed as a potential conflict of interest.

## Publisher’s note

All claims expressed in this article are solely those of the authors and do not necessarily represent those of their affiliated organizations, or those of the publisher, the editors and the reviewers. Any product that may be evaluated in this article, or claim that may be made by its manufacturer, is not guaranteed or endorsed by the publisher.
